# Supplementary material for: Consumers’ Understanding of Ultra-Processed Foods
Source: Foods. 2022 May 7;11(9):1359. doi: 10.3390/foods11091359 (PMC9099562; doi:10.3390/foods11091359)
Supplement: Supplementary file 1 [file foods-11-01359-s001.zip › foods-1693622-supplementary.pdf]

**Respondent ID**	**iz Summary**			**Quiz Results**				
	**Score**	**Points**	**Percentile**	**Correct**	**Incorrect**	**Total_Questions**	**Do you know what is ultra-processed food?**	**Did you already know this definition?**
	80%	8/10	88%	8	2	10	Yes	No
	50%	5/10	27%	5	5	10	Yes	No
	70%	7/10	68%	7	3	10	Yes	No
	60%	6/10	46%	6	4	10	Yes	Yes
	90%	9/10	99%	9	1	10	Yes	No
	50%	5/10	27%	5	5	10	Yes	Yes
	40%	4/10	12%	4	6	10	Yes	No
	50%	5/10	27%	5	5	10	Yes	No
	80%	8/10	88%	8	2	10	Yes	No
	80%	8/10	88%	8	2	10	Yes	No
	40%	4/10	12%	4	6	10	No	No
	60%	6/10	46%	6	4	10	Yes	No
	80%	8/10	88%	8	2	10	Yes	Yes
	60%	6/10	46%	6	4	10	Yes	No
	90%	9/10	99%	9	1	10	Yes	Yes
	90%	9/10	99%	9	1	10	Yes	No
	60%	6/10	46%	6	4	10	Yes	Yes
	50%	5/10	27%	5	5	10	Yes	No
	50%	5/10	27%	5	5	10	Yes	No
	60%	6/10	46%	6	4	10	No	No
	50%	5/10	27%	5	5	10	Yes	No
	80%	8/10	88%	8	2	10	Yes	Yes
	50%	5/10	27%	5	5	10	No	No
	90%	9/10	99%	9	1	10	No	No
	40%	4/10	12%	4	6	10	Yes	Yes
	60%	6/10	46%	6	4	10	Yes	No
	50%	5/10	27%	5	5	10	Yes	No
	70%	7/10	68%	7	3	10	Yes	Yes
	50%	5/10	27%	5	5	10	Yes	No
	80%	8/10	88%	8	2	10	Yes	No
	90%	9/10	99%	9	1	10	Yes	No
	50%	5/10	27%	5	5	10	Yes	No
	70%	7/10	68%	7	3	10	Yes	No
	90%	9/10	99%	9	1	10	Yes	No
	60%	6/10	46%	6	4	10	Yes	Yes
	50%	5/10	27%	5	5	10	Yes	Yes
	60%	6/10	46%	6	4	10	Yes	Yes
	80%	8/10	88%	8	2	10	Yes	No
	90%	9/10	99%	9	1	10	Yes	No
	80%	8/10	88%	8	2	10	No	No
	70%	7/10	68%	7	3	10	Yes	No
	50%	5/10	27%	5	5	10	Yes	No
	50%	5/10	27%	5	5	10	Yes	Yes
	70%	7/10	68%	7	3	10	Yes	No
	30%	3/10	4%	3	7	10	Yes	No

Respondent ID	iz Summary		Quiz Results					
	Score	Points	Percentile	Correct	Incorrect	Total_Questions	Do you know what is ultra-processed food?	Did you already know this definition?
	90%	9/10	99%	9	1	10	Yes	Yes
	30%	3/10	4%	3	7	10	Yes	No
	90%	9/10	99%	9	1	10	Yes	Yes
	80%	8/10	88%	8	2	10	Yes	Yes
	60%	6/10	46%	6	4	10	Yes	No
	90%	9/10	99%	9	1	10	Yes	No
	80%	8/10	88%	8	2	10	Yes	No
	50%	5/10	27%	5	5	10	Yes	Yes
	60%	6/10	46%	6	4	10	Yes	Yes
	60%	6/10	46%	6	4	10	Yes	No
	60%	6/10	46%	6	4	10	Yes	No
	50%	5/10	27%	5	5	10	Yes	No
	60%	6/10	46%	6	4	10	Yes	Yes
	60%	6/10	46%	6	4	10	Yes	Yes
	50%	5/10	27%	5	5	10	Yes	Yes
	80%	8/10	88%	8	2	10	Yes	No
	40%	4/10	12%	4	6	10	Yes	No
	70%	7/10	68%	7	3	10	Yes	Yes
	70%	7/10	68%	7	3	10	Yes	No
	70%	7/10	68%	7	3	10	Yes	No
	50%	5/10	27%	5	5	10	Yes	Yes
	60%	6/10	46%	6	4	10	Yes	Yes
	70%	7/10	68%	7	3	10	Yes	Yes
	80%	8/10	88%	8	2	10	Yes	No
	80%	8/10	88%	8	2	10	Yes	No
	80%	8/10	88%	8	2	10	Yes	No
	60%	6/10	46%	6	4	10	No	No
	80%	8/10	88%	8	2	10	Yes	Yes
	50%	5/10	27%	5	5	10	No	No
	70%	7/10	68%	7	3	10	Yes	Yes
	60%	6/10	46%	6	4	10	No	No
	70%	7/10	68%	7	3	10	Yes	No
	40%	4/10	12%	4	6	10	No	No
	60%	6/10	46%	6	4	10	No	No
	60%	6/10	46%	6	4	10	No	No
	70%	7/10	68%	7	3	10	Yes	Yes
	80%	8/10	88%	8	2	10	Yes	Yes
	80%	8/10	88%	8	2	10	Yes	No
	70%	7/10	68%	7	3	10	Yes	No
	90%	9/10	99%	9	1	10	Yes	Yes
	50%	5/10	27%	5	5	10	No	No
	80%	8/10	88%	8	2	10	Yes	No
	50%	5/10	27%	5	5	10	Yes	No
	60%	6/10	46%	6	4	10	Yes	No
	60%	6/10	46%	6	4	10	Yes	No

**Respondent ID**	**iz Summary**			**Quiz Results**				
	**Score**	**Points**	**Percentile**	**Correct**	**Incorrect**	**Total_Questions**	**Do you know what is ultra-processed food?**	**Did you already know this definition?**
	60%	6/10	46%	6	4	10	Yes	Yes
	60%	6/10	46%	6	4	10	Yes	No
	60%	6/10	46%	6	4	10	Yes	No
	90%	9/10	99%	9	1	10	Yes	Yes
	50%	5/10	27%	5	5	10	Yes	No
	60%	6/10	46%	6	4	10	Yes	No
	90%	9/10	99%	9	1	10	Yes	No
	70%	7/10	68%	7	3	10	Yes	No
	80%	8/10	88%	8	2	10	Yes	No
	70%	7/10	68%	7	3	10	No	No
	90%	9/10	99%	9	1	10	Yes	No
	70%	7/10	68%	7	3	10	No	No
	80%	8/10	88%	8	2	10	Yes	No
	90%	9/10	99%	9	1	10	Yes	No
	50%	5/10	27%	5	5	10	Yes	No
	40%	4/10	12%	4	6	10	Yes	No
	70%	7/10	68%	7	3	10	Yes	No
	80%	8/10	88%	8	2	10	No	No
	50%	5/10	27%	5	5	10	Yes	Yes
	80%	8/10	88%	8	2	10	Yes	Yes
	60%	6/10	46%	6	4	10	Yes	No
	90%	9/10	99%	9	1	10	Yes	Yes
	90%	9/10	99%	9	1	10	Yes	Yes
	70%	7/10	68%	7	3	10	Yes	Yes
	90%	9/10	99%	9	1	10	Yes	Yes
	70%	7/10	68%	7	3	10	Yes	No
	80%	8/10	88%	8	2	10	Yes	Yes
	60%	6/10	46%	6	4	10	No	No
	80%	8/10	88%	8	2	10	Yes	No
	70%	7/10	68%	7	3	10	Yes	No
	80%	8/10	88%	8	2	10	Yes	No
	50%	5/10	27%	5	5	10	No	No
	60%	6/10	46%	6	4	10	Yes	No
	40%	4/10	12%	4	6	10	No	No
	40%	4/10	12%	4	6	10	Yes	No
	90%	9/10	99%	9	1	10	No	No
	70%	7/10	68%	7	3	10	Yes	Yes
	90%	9/10	99%	9	1	10	Yes	No
	70%	7/10	68%	7	3	10	No	No
	60%	6/10	46%	6	4	10	No	No
	80%	8/10	88%	8	2	10	No	No
	70%	7/10	68%	7	3	10	Yes	No
	80%	8/10	88%	8	2	10	No	No
	50%	5/10	27%	5	5	10	No	No
	60%	6/10	46%	6	4	10	Yes	No

Respondent ID	iz Summary			Quiz Results				
	Score	Points	Percentile	Correct	Incorrect	Total_Questions	Do you know what is ultra-processed food?	Did you already know this definition?
	50%	5/10	27%	5	5	10	Yes	No
	90%	9/10	99%	9	1	10	Yes	Yes
	70%	7/10	68%	7	3	10	Yes	Yes
	40%	4/10	12%	4	6	10	Yes	No
	90%	9/10	99%	9	1	10	No	No
	60%	6/10	46%	6	4	10	Yes	Yes
	70%	7/10	68%	7	3	10	Yes	No
	40%	4/10	12%	4	6	10	No	No
	60%	6/10	46%	6	4	10	No	No
	90%	9/10	99%	9	1	10	Yes	Yes
	70%	7/10	68%	7	3	10	Yes	No
	70%	7/10	68%	7	3	10	No	No
	50%	5/10	27%	5	5	10	No	No
	50%	5/10	27%	5	5	10	Yes	No
	70%	7/10	68%	7	3	10	Yes	No
	50%	5/10	27%	5	5	10	Yes	No
	60%	6/10	46%	6	4	10	Yes	No
	80%	8/10	88%	8	2	10	Yes	Yes
	60%	6/10	46%	6	4	10	Yes	No
	80%	8/10	88%	8	2	10	No	No
	70%	7/10	68%	7	3	10	No	No
	30%	3/10	4%	3	7	10	Yes	No
	70%	7/10	68%	7	3	10	Yes	No
	50%	5/10	27%	5	5	10	No	No
	60%	6/10	46%	6	4	10	Yes	No
	70%	7/10	68%	7	3	10	No	No
	60%	6/10	46%	6	4	10	No	No
	60%	6/10	46%	6	4	10	Yes	Yes
	90%	9/10	99%	9	1	10	Yes	Yes
	80%	8/10	88%	8	2	10	Yes	No
	40%	4/10	12%	4	6	10	Yes	No
	80%	8/10	88%	8	2	10	Yes	Yes
	70%	7/10	68%	7	3	10	Yes	Yes
	60%	6/10	46%	6	4	10	Yes	No
	80%	8/10	88%	8	2	10	Yes	Yes
	60%	6/10	46%	6	4	10	Yes	Yes
	60%	6/10	46%	6	4	10	Yes	No
	80%	8/10	88%	8	2	10	Yes	No
	60%	6/10	46%	6	4	10	Yes	No
	80%	8/10	88%	8	2	10	Yes	No
	50%	5/10	27%	5	5	10	Yes	No
	70%	7/10	68%	7	3	10	Yes	No
	60%	6/10	46%	6	4	10	Yes	No
	70%	7/10	68%	7	3	10	No	No
	60%	6/10	46%	6	4	10	Yes	No

Respondent ID	iz Summary		Quiz Results					
	Score	Points	Percentile	Correct	Incorrect	Total_Questions	Do you know what is ultra-processed food?	Did you already know this definition?
	50%	5/10	27%	5	5	10	Yes	No
	50%	5/10	27%	5	5	10	Yes	No
	60%	6/10	46%	6	4	10	Yes	No
	80%	8/10	88%	8	2	10	No	No
	40%	4/10	12%	4	6	10	Yes	No
	40%	4/10	12%	4	6	10	Yes	No
	80%	8/10	88%	8	2	10	Yes	No
	60%	6/10	46%	6	4	10	Yes	No
	70%	7/10	68%	7	3	10	Yes	No
	70%	7/10	68%	7	3	10	Yes	Yes
	50%	5/10	27%	5	5	10	No	No
	80%	8/10	88%	8	2	10	No	No
	60%	6/10	46%	6	4	10	Yes	No
	70%	7/10	68%	7	3	10	Yes	No
	80%	8/10	88%	8	2	10	Yes	No
	100%	10/10	100%	10	0	10	Yes	Yes
	70%	7/10	68%	7	3	10	Yes	No
	70%	7/10	68%	7	3	10	Yes	Yes
	50%	5/10	27%	5	5	10	Yes	No
	70%	7/10	68%	7	3	10	Yes	No
	90%	9/10	99%	9	1	10	Yes	No
	70%	7/10	68%	7	3	10	Yes	No
	40%	4/10	12%	4	6	10	Yes	No
	80%	8/10	88%	8	2	10	Yes	No
	70%	7/10	68%	7	3	10	Yes	Yes
	40%	4/10	12%	4	6	10	No	No
	70%	7/10	68%	7	3	10	Yes	Yes
	60%	6/10	46%	6	4	10	Yes	No
	70%	7/10	68%	7	3	10	Yes	No
	70%	7/10	68%	7	3	10	Yes	No
	60%	6/10	46%	6	4	10	Yes	No
	50%	5/10	27%	5	5	10	Yes	Yes
	50%	5/10	27%	5	5	10	Yes	No
	50%	5/10	27%	5	5	10	Yes	No
	90%	9/10	99%	9	1	10	Yes	Yes
	70%	7/10	68%	7	3	10	Yes	No
	50%	5/10	27%	5	5	10	Yes	No
	60%	6/10	46%	6	4	10	Yes	No
	80%	8/10	88%	8	2	10	Yes	Yes
	60%	6/10	46%	6	4	10	No	No
	60%	6/10	46%	6	4	10	Yes	Yes
	90%	9/10	99%	9	1	10	Yes	No
	50%	5/10	27%	5	5	10	Yes	No
	50%	5/10	27%	5	5	10	No	No
	80%	8/10	88%	8	2	10	Yes	No

Respondent ID	iz Summary			Quiz Results				
	Score	Points	Percentile	Correct	Incorrect	Total_Questions	Do you know what is ultra-processed food?	Did you already know this definition?
	70%	7/10	68%	7	3	10	No	No
	80%	8/10	88%	8	2	10	Yes	No
	40%	4/10	12%	4	6	10	No	No
	60%	6/10	46%	6	4	10	Yes	No
	60%	6/10	46%	6	4	10	Yes	Yes
	70%	7/10	68%	7	3	10	Yes	No
	50%	5/10	27%	5	5	10	Yes	Yes
	40%	4/10	12%	4	6	10	Yes	No
	80%	8/10	88%	8	2	10	Yes	No
	50%	5/10	27%	5	5	10	Yes	No
	50%	5/10	27%	5	5	10	Yes	No
	50%	5/10	27%	5	5	10	Yes	No
	50%	5/10	27%	5	5	10	Yes	No
	70%	7/10	68%	7	3	10	No	No
	40%	4/10	12%	4	6	10	Yes	Yes
	60%	6/10	46%	6	4	10	No	No
	60%	6/10	46%	6	4	10	Yes	Yes
	90%	9/10	99%	9	1	10	Yes	Yes
	60%	6/10	46%	6	4	10	Yes	Yes
	60%	6/10	46%	6	4	10	Yes	No
	80%	8/10	88%	8	2	10	Yes	No
	80%	8/10	88%	8	2	10	Yes	No
	60%	6/10	46%	6	4	10	Yes	No
	80%	8/10	88%	8	2	10	Yes	Yes
	60%	6/10	46%	6	4	10	Yes	Yes
	90%	9/10	99%	9	1	10	Yes	No
	80%	8/10	88%	8	2	10	Yes	No
	80%	8/10	88%	8	2	10	Yes	No
	60%	6/10	46%	6	4	10	Yes	No
	90%	9/10	99%	9	1	10	Yes	No
	80%	8/10	88%	8	2	10	No	No
	80%	8/10	88%	8	2	10	Yes	No
	80%	8/10	88%	8	2	10	Yes	Yes
	50%	5/10	27%	5	5	10	Yes	No
	70%	7/10	68%	7	3	10	Yes	Yes
	70%	7/10	68%	7	3	10	Yes	Yes
	70%	7/10	68%	7	3	10	Yes	No
	70%	7/10	68%	7	3	10	Yes	No
	80%	8/10	88%	8	2	10	Yes	Yes
	80%	8/10	88%	8	2	10	Yes	Yes
	30%	3/10	4%	3	7	10	Yes	No
	40%	4/10	12%	4	6	10	No	No
	40%	4/10	12%	4	6	10	Yes	Yes
	80%	8/10	88%	8	2	10	Yes	Yes
	60%	6/10	46%	6	4	10	Yes	Yes

Respondent ID	iz Summary		Quiz Results					
	Score	Points	Percentile	Correct	Incorrect	Total_Questions	Do you know what is ultra-processed food?	Did you already know this definition?
	50%	5/10	27%	5	5	10	No	No
	50%	5/10	27%	5	5	10	Yes	Yes
	40%	4/10	12%	4	6	10	No	No
	70%	7/10	68%	7	3	10	Yes	No
	40%	4/10	12%	4	6	10	Yes	No
	80%	8/10	88%	8	2	10	Yes	No
	80%	8/10	88%	8	2	10	No	No
	80%	8/10	88%	8	2	10	Yes	Yes
	70%	7/10	68%	7	3	10	Yes	Yes
	50%	5/10	27%	5	5	10	Yes	Yes
	70%	7/10	68%	7	3	10	Yes	Yes
	90%	9/10	99%	9	1	10	Yes	No
	60%	6/10	46%	6	4	10	Yes	No
	50%	5/10	27%	5	5	10	Yes	No
	40%	4/10	12%	4	6	10	Yes	No
	70%	7/10	68%	7	3	10	Yes	No
	60%	6/10	46%	6	4	10	Yes	No
	30%	3/10	4%	3	7	10	Yes	Yes
	30%	3/10	4%	3	7	10	Yes	Yes
	70%	7/10	68%	7	3	10	Yes	No
	70%	7/10	68%	7	3	10	Yes	No
	70%	7/10	68%	7	3	10	No	No
	60%	6/10	46%	6	4	10	Yes	No
	80%	8/10	88%	8	2	10	Yes	Yes
	80%	8/10	88%	8	2	10	Yes	Yes
	70%	7/10	68%	7	3	10	Yes	Yes
	40%	4/10	12%	4	6	10	Yes	Yes
	60%	6/10	46%	6	4	10	Yes	No
	70%	7/10	68%	7	3	10	Yes	Yes
	70%	7/10	68%	7	3	10	Yes	No
	100%	10/10	100%	10	0	10	Yes	Yes
	70%	7/10	68%	7	3	10	Yes	No
	80%	8/10	88%	8	2	10	Yes	Yes
	60%	6/10	46%	6	4	10	Yes	No
	80%	8/10	88%	8	2	10	Yes	Yes
	70%	7/10	68%	7	3	10	Yes	No
	90%	9/10	99%	9	1	10	Yes	Yes
	90%	9/10	99%	9	1	10	Yes	Yes
	90%	9/10	99%	9	1	10	Yes	Yes
	80%	8/10	88%	8	2	10	Yes	No
	80%	8/10	88%	8	2	10	Yes	Yes
	80%	8/10	88%	8	2	10	Yes	Yes
	80%	8/10	88%	8	2	10	Yes	Yes
	50%	5/10	27%	5	5	10	Yes	Yes
	50%	5/10	27%	5	5	10	Yes	No

**Respondent ID**	**iz Summary**			**Quiz Results**				
	**Score**	**Points**	**Percentile**	**Correct**	**Incorrect**	**Total_Questions**	**Do you know what is ultra-processed food?**	**Did you already know this definition?**
	70%	7/10	68%	7	3	10	Yes	Yes
	80%	8/10	88%	8	2	10	Yes	Yes
	90%	9/10	99%	9	1	10	Yes	Yes
	90%	9/10	99%	9	1	10	Yes	Yes
	70%	7/10	68%	7	3	10	Yes	Yes
	80%	8/10	88%	8	2	10	Yes	Yes
	70%	7/10	68%	7	3	10	Yes	No
	50%	5/10	27%	5	5	10	Yes	No
	80%	8/10	88%	8	2	10	Yes	Yes
	70%	7/10	68%	7	3	10	Yes	Yes
	80%	8/10	88%	8	2	10	Yes	Yes
	90%	9/10	99%	9	1	10	Yes	Yes
	80%	8/10	88%	8	2	10	No	No
	80%	8/10	88%	8	2	10	Yes	No
	70%	7/10	68%	7	3	10	Yes	No
	80%	8/10	88%	8	2	10	Yes	No
	80%	8/10	88%	8	2	10	Yes	No
	70%	7/10	68%	7	3	10	No	No
	50%	5/10	27%	5	5	10	Yes	No
	60%	6/10	46%	6	4	10	Yes	No
	80%	8/10	88%	8	2	10	No	No
	60%	6/10	46%	6	4	10	Yes	Yes
	60%	6/10	46%	6	4	10	Yes	No
	50%	5/10	27%	5	5	10	Yes	Yes
	70%	7/10	68%	7	3	10	Yes	No
	70%	7/10	68%	7	3	10	Yes	No
	60%	6/10	46%	6	4	10	No	No
	80%	8/10	88%	8	2	10	Yes	No
	70%	7/10	68%	7	3	10	Yes	No
	80%	8/10	88%	8	2	10	Yes	No
	70%	7/10	68%	7	3	10	Yes	No
	70%	7/10	68%	7	3	10	Yes	No
	90%	9/10	99%	9	1	10	Yes	No
	50%	5/10	27%	5	5	10	Yes	No
	60%	6/10	46%	6	4	10	No	No
	70%	7/10	68%	7	3	10	Yes	Yes
	80%	8/10	88%	8	2	10	No	No
	60%	6/10	46%	6	4	10	Yes	No
	60%	6/10	46%	6	4	10	Yes	Yes
	70%	7/10	68%	7	3	10	Yes	No
	20%	2/10	1%	2	8	10	Yes	No
	70%	7/10	68%	7	3	10	Yes	Yes
	70%	7/10	68%	7	3	10	Yes	Yes
	80%	8/10	88%	8	2	10	Yes	No
	80%	8/10	88%	8	2	10	Yes	Yes

**Respondent ID**	**iz Summary**			**Quiz Results**				
	**Score**	**Points**	**Percentile**	**Correct**	**Incorrect**	**Total_Questions**	**Do you know what is ultra-processed food?**	**Did you already know this definition?**
	60%	6/10	46%	6	4	10	Yes	No
	80%	8/10	88%	8	2	10	Yes	Yes
	50%	5/10	27%	5	5	10	Yes	No
	70%	7/10	68%	7	3	10	No	No
	20%	2/10	1%	2	8	10	Yes	Yes
	40%	4/10	12%	4	6	10	Yes	No
	70%	7/10	68%	7	3	10	Yes	Yes
	50%	5/10	27%	5	5	10	Yes	Yes
	70%	7/10	68%	7	3	10	Yes	No
	30%	3/10	4%	3	7	10	Yes	Yes
	70%	7/10	68%	7	3	10	Yes	Yes
	70%	7/10	68%	7	3	10	Yes	Yes
	80%	8/10	88%	8	2	10	Yes	Yes
	80%	8/10	88%	8	2	10	Yes	No
	50%	5/10	27%	5	5	10	Yes	Yes
	40%	4/10	12%	4	6	10	Yes	No
	70%	7/10	68%	7	3	10	Yes	No
	60%	6/10	46%	6	4	10	Yes	Yes
	70%	7/10	68%	7	3	10	Yes	No
	90%	9/10	99%	9	1	10	Yes	Yes
	80%	8/10	88%	8	2	10	Yes	No
	100%	10/10	100%	10	0	10	No	No
	70%	7/10	68%	7	3	10	No	No
	50%	5/10	27%	5	5	10	Yes	Yes
	30%	3/10	4%	3	7	10	Yes	No
	60%	6/10	46%	6	4	10	Yes	No
	40%	4/10	12%	4	6	10	No	No
	50%	5/10	27%	5	5	10	Yes	Yes
	50%	5/10	27%	5	5	10	No	No
	60%	6/10	46%	6	4	10	Yes	No
	60%	6/10	46%	6	4	10	Yes	No
	80%	8/10	88%	8	2	10	Yes	Yes
	40%	4/10	12%	4	6	10	Yes	No
	90%	9/10	99%	9	1	10	Yes	No
	80%	8/10	88%	8	2	10	Yes	No
	90%	9/10	99%	9	1	10	Yes	Yes
	90%	9/10	99%	9	1	10	Yes	Yes
	30%	3/10	4%	3	7	10	Yes	No
	70%	7/10	68%	7	3	10	Yes	No
	60%	6/10	46%	6	4	10	No	No
	90%	9/10	99%	9	1	10	Yes	Yes
	90%	9/10	99%	9	1	10	Yes	No
	50%	5/10	27%	5	5	10	Yes	Yes
	70%	7/10	68%	7	3	10	No	No
	50%	5/10	27%	5	5	10	Yes	No

**Respondent ID**	**iz Summary**		**Quiz Results**					
	**Score**	**Points**	**Percentile**	**Correct**	**Incorrect**	**Total_Questions**	**Do you know what is ultra-processed food?**	**Did you already know this definition?**
	50%	5/10	27%	5	5	10	No	No
	60%	6/10	46%	6	4	10	Yes	Yes
	60%	6/10	46%	6	4	10	Yes	No
	80%	8/10	88%	8	2	10	Yes	Yes
	70%	7/10	68%	7	3	10	No	No
	70%	7/10	68%	7	3	10	Yes	Yes
	60%	6/10	46%	6	4	10	Yes	No
	40%	4/10	12%	4	6	10	Yes	No
	50%	5/10	27%	5	5	10	Yes	No
	80%	8/10	88%	8	2	10	Yes	No
	60%	6/10	46%	6	4	10	Yes	No
	50%	5/10	27%	5	5	10	Yes	No
	60%	6/10	46%	6	4	10	No	No
	50%	5/10	27%	5	5	10	Yes	Yes
	60%	6/10	46%	6	4	10	Yes	No
	50%	5/10	27%	5	5	10	Yes	Yes
	50%	5/10	27%	5	5	10	Yes	Yes
	90%	9/10	99%	9	1	10	Yes	Yes
	60%	6/10	46%	6	4	10	Yes	No
	80%	8/10	88%	8	2	10	Yes	No
	80%	8/10	88%	8	2	10	Yes	No
	30%	3/10	4%	3	7	10	Yes	Yes
	80%	8/10	88%	8	2	10	Yes	Yes
	40%	4/10	12%	4	6	10	Yes	Yes
	40%	4/10	12%	4	6	10	Yes	Yes
	70%	7/10	68%	7	3	10	Yes	Yes
	80%	8/10	88%	8	2	10	Yes	Yes
	60%	6/10	46%	6	4	10	Yes	Yes
	70%	7/10	68%	7	3	10	Yes	No
	70%	7/10	68%	7	3	10	Yes	Yes
	50%	5/10	27%	5	5	10	Yes	Yes
	70%	7/10	68%	7	3	10	No	No
	70%	7/10	68%	7	3	10	Yes	Yes
	70%	7/10	68%	7	3	10	Yes	No
	90%	9/10	99%	9	1	10	No	No
	60%	6/10	46%	6	4	10	Yes	No
	60%	6/10	46%	6	4	10	Yes	No
	70%	7/10	68%	7	3	10	No	No
	90%	9/10	99%	9	1	10	Yes	No
	80%	8/10	88%	8	2	10	No	No
	60%	6/10	46%	6	4	10	Yes	No
	60%	6/10	46%	6	4	10	Yes	Yes
	40%	4/10	12%	4	6	10	Yes	Yes
	80%	8/10	88%	8	2	10	Yes	No
	40%	4/10	12%	4	6	10	No	No

Respondent ID	iz Summary		Quiz Results					
	Score	Points	Percentile	Correct	Incorrect	Total_Questions	Do you know what is ultra-processed food?	Did you already know this definition?
	40%	4/10	12%	4	6	10	Yes	No
	30%	3/10	4%	3	7	10	Yes	Yes
	50%	5/10	27%	5	5	10	Yes	Yes
	60%	6/10	46%	6	4	10	Yes	No
	70%	7/10	68%	7	3	10	Yes	Yes
	80%	8/10	88%	8	2	10	Yes	Yes
	80%	8/10	88%	8	2	10	Yes	Yes
	70%	7/10	68%	7	3	10	Yes	Yes
	60%	6/10	46%	6	4	10	Yes	No
	80%	8/10	88%	8	2	10	No	No
	30%	3/10	4%	3	7	10	Yes	Yes
	70%	7/10	68%	7	3	10	Yes	Yes
	80%	8/10	88%	8	2	10	Yes	Yes
	60%	6/10	46%	6	4	10	No	No
	80%	8/10	88%	8	2	10	Yes	Yes
	70%	7/10	68%	7	3	10	Yes	No
	90%	9/10	99%	9	1	10	Yes	Yes
	60%	6/10	46%	6	4	10	Yes	Yes
	40%	4/10	12%	4	6	10	No	No
	50%	5/10	27%	5	5	10	Yes	Yes
	70%	7/10	68%	7	3	10	No	No
	70%	7/10	68%	7	3	10	Yes	No
	60%	6/10	46%	6	4	10	No	No
	60%	6/10	46%	6	4	10	No	No
	40%	4/10	12%	4	6	10	Yes	Yes
	70%	7/10	68%	7	3	10	Yes	Yes
	70%	7/10	68%	7	3	10	Yes	Yes
	60%	6/10	46%	6	4	10	Yes	Yes
	80%	8/10	88%	8	2	10	No	No
	50%	5/10	27%	5	5	10	No	No
	60%	6/10	46%	6	4	10	Yes	No
	60%	6/10	46%	6	4	10	Yes	Yes
	60%	6/10	46%	6	4	10	Yes	No
	60%	6/10	46%	6	4	10	Yes	No
	40%	4/10	12%	4	6	10	No	No
	70%	7/10	68%	7	3	10	Yes	No
	70%	7/10	68%	7	3	10	Yes	Yes
	70%	7/10	68%	7	3	10	Yes	No
	20%	2/10	1%	2	8	10	Yes	Yes
	70%	7/10	68%	7	3	10	No	No
	30%	3/10	4%	3	7	10	Yes	No
	30%	3/10	4%	3	7	10	Yes	No
	30%	3/10	4%	3	7	10	Yes	Yes
	60%	6/10	46%	6	4	10	Yes	No
	50%	5/10	27%	5	5	10	No	No

**Respondent ID**	**iz Summary**			**Quiz Results**				
	**Score**	**Points**	**Percentile**	**Correct**	**Incorrect**	**Total_Questions**	**Do you know what is ultra-processed food?**	**Did you already know this definition?**
	30%	3/10	4%	3	7	10	Yes	Yes
	80%	8/10	88%	8	2	10	Yes	No
	40%	4/10	12%	4	6	10	Yes	No
	60%	6/10	46%	6	4	10	Yes	Yes
	40%	4/10	12%	4	6	10	Yes	No
	90%	9/10	99%	9	1	10	Yes	No
	40%	4/10	12%	4	6	10	No	No
	20%	2/10	1%	2	8	10	Yes	No
	40%	4/10	12%	4	6	10	Yes	No
	60%	6/10	46%	6	4	10	Yes	Yes
	70%	7/10	68%	7	3	10	Yes	Yes
	60%	6/10	46%	6	4	10	Yes	Yes
	70%	7/10	68%	7	3	10	Yes	No
	70%	7/10	68%	7	3	10	Yes	Yes
	50%	5/10	27%	5	5	10	No	No
	50%	5/10	27%	5	5	10	Yes	No
	80%	8/10	88%	8	2	10	Yes	No
	70%	7/10	68%	7	3	10	Yes	Yes
	70%	7/10	68%	7	3	10	Yes	No
	80%	8/10	88%	8	2	10	Yes	No
	60%	6/10	46%	6	4	10	Yes	No
	70%	7/10	68%	7	3	10	Yes	No
	50%	5/10	27%	5	5	10	No	No
	40%	4/10	12%	4	6	10	No	No
	70%	7/10	68%	7	3	10	Yes	No
	80%	8/10	88%	8	2	10	No	No
	70%	7/10	68%	7	3	10	Yes	No
	80%	8/10	88%	8	2	10	Yes	Yes
	90%	9/10	99%	9	1	10	No	No
	80%	8/10	88%	8	2	10	Yes	No
	80%	8/10	88%	8	2	10	Yes	No
	70%	7/10	68%	7	3	10	Yes	No
	90%	9/10	99%	9	1	10	Yes	No
	80%	8/10	88%	8	2	10	Yes	No
	80%	8/10	88%	8	2	10	Yes	No
	70%	7/10	68%	7	3	10	Yes	No
	90%	9/10	99%	9	1	10	Yes	Yes
	100%	10/10	100%	10	0	10	Yes	Yes
	90%	9/10	99%	9	1	10	Yes	Yes
	90%	9/10	99%	9	1	10	Yes	Yes
	100%	10/10	100%	10	0	10	Yes	Yes
	50%	5/10	27%	5	5	10	Yes	Yes
	50%	5/10	27%	5	5	10	Yes	No
	90%	9/10	99%	9	1	10	Yes	Yes
	90%	9/10	99%	9	1	10	Yes	Yes

Respondent ID	iz Summary			Quiz Results				
	Score	Points	Percentile	Correct	Incorrect	Total_Questions	Do you know what is ultra-processed food?	Did you already know this definition?
	60%	6/10	46%	6	4	10	Yes	Yes
	100%	10/10	100%	10	0	10	Yes	Yes
	70%	7/10	68%	7	3	10	Yes	Yes
	80%	8/10	88%	8	2	10	Yes	Yes
	50%	5/10	25%	5	5	10	Yes	No
	60%	6/10	46%	6	4	10	Yes	Yes
	80%	8/10	89%	8	2	10	No	No
	70%	7/10	68%	7	3	10	Yes	No
	80%	8/10	89%	8	2	10	No	No
	40%	4/10	10%	4	6	10	Yes	No
	80%	8/10	89%	8	2	10	Yes	Yes
	70%	7/10	68%	7	3	10	Yes	Yes
	60%	6/10	46%	6	4	10	Yes	No
	40%	4/10	10%	4	6	10	Yes	Yes
	70%	7/10	68%	7	3	10	No	No
	60%	6/10	46%	6	4	10	No	No
	60%	6/10	46%	6	4	10	Yes	No
	40%	4/10	10%	4	6	10	Yes	No
	70%	7/10	68%	7	3	10	Yes	No
	100%	10/10	100%	10	0	10	Yes	Yes
	70%	7/10	68%	7	3	10	No	No
	60%	6/10	46%	6	4	10	Yes	No
	50%	5/10	25%	5	5	10	Yes	No
	70%	7/10	68%	7	3	10	Yes	Yes
	80%	8/10	89%	8	2	10	Yes	No
	50%	5/10	25%	5	5	10	Yes	Yes
	90%	9/10	99%	9	1	10	Yes	Yes
	60%	6/10	46%	6	4	10	No	No
	50%	5/10	25%	5	5	10	No	Yes
	70%	7/10	68%	7	3	10	Yes	Yes
	30%	3/10	3%	3	7	10	No	No
	80%	8/10	89%	8	2	10	Yes	No
	50%	5/10	25%	5	5	10	No	No
	50%	5/10	25%	5	5	10	Yes	No
	90%	9/10	99%	9	1	10	Yes	Yes
	60%	6/10	46%	6	4	10	Yes	No
	80%	8/10	89%	8	2	10	Yes	Yes
	50%	5/10	25%	5	5	10	Yes	No
	80%	8/10	89%	8	2	10	Yes	No
	40%	4/10	10%	4	6	10	No	No
	80%	8/10	89%	8	2	10	Yes	No
	50%	5/10	25%	5	5	10	No	No
	80%	8/10	89%	8	2	10	Yes	Yes
	70%	7/10	68%	7	3	10	No	No
	80%	8/10	89%	8	2	10	Yes	Yes

Respondent ID	iz Summary		Quiz Results					
	Score	Points	Percentile	Correct	Incorrect	Total_Questions	Do you know what is ultra-processed food?	Did you already know this definition?
	60%	6/10	46%	6	4	10	Yes	No
	60%	6/10	46%	6	4	10	Yes	No
	40%	4/10	10%	4	6	10	No	No
	80%	8/10	89%	8	2	10	Yes	Yes
	50%	5/10	25%	5	5	10	Yes	No
	70%	7/10	68%	7	3	10	Yes	Yes
	80%	8/10	89%	8	2	10	Yes	No
	60%	6/10	46%	6	4	10	Yes	No
	80%	8/10	89%	8	2	10	Yes	Yes
	60%	6/10	46%	6	4	10	No	No
	60%	6/10	46%	6	4	10	No	No
	80%	8/10	89%	8	2	10	No	No
	50%	5/10	25%	5	5	10	Yes	No
	0%	0/10	0%	0	10	10	Yes	Yes
	50%	5/10	25%	5	5	10	No	No
	50%	5/10	25%	5	5	10	Yes	Yes
	50%	5/10	25%	5	5	10	Yes	No
	60%	6/10	46%	6	4	10	Yes	Yes
	60%	6/10	46%	6	4	10	Yes	No
	60%	6/10	46%	6	4	10	No	No
	60%	6/10	46%	6	4	10	No	No
	40%	4/10	10%	4	6	10	Yes	Yes
	80%	8/10	89%	8	2	10	Yes	No
	50%	5/10	25%	5	5	10	Yes	No
	60%	6/10	46%	6	4	10	Yes	No
	70%	7/10	68%	7	3	10	No	No
	90%	9/10	99%	9	1	10	Yes	Yes
	70%	7/10	68%	7	3	10	Yes	No
	70%	7/10	68%	7	3	10	Yes	No
	60%	6/10	46%	6	4	10	Yes	No
	70%	7/10	68%	7	3	10	Yes	No
	50%	5/10	25%	5	5	10	Yes	No
	80%	8/10	89%	8	2	10	No	No
	70%	7/10	68%	7	3	10	Yes	Yes
	80%	8/10	89%	8	2	10	No	No
	60%	6/10	46%	6	4	10	Yes	Yes
	60%	6/10	46%	6	4	10	Yes	No
	90%	9/10	99%	9	1	10	Yes	No
	80%	8/10	89%	8	2	10	No	No
	70%	7/10	68%	7	3	10	Yes	Yes
	60%	6/10	46%	6	4	10	Yes	No
	30%	3/10	3%	3	7	10	Yes	Yes
	30%	3/10	3%	3	7	10	Yes	Yes
	60%	6/10	46%	6	4	10	Yes	Yes
	80%	8/10	89%	8	2	10	Yes	No

Respondent ID	iz Summary Score	Points	Quiz Results Percentile	Correct	Incorrect	Total_Questions	Do you know what is ultra-processed food?	Did you already know this definition?
	80%	8/10	89%	8	2	10	Yes	Yes
	60%	6/10	46%	6	4	10	Yes	No
	80%	8/10	89%	8	2	10	Yes	Yes
	70%	7/10	68%	7	3	10	Yes	No
	60%	6/10	46%	6	4	10	Yes	No
	70%	7/10	68%	7	3	10	Yes	Yes
	20%	2/10	1%	2	8	10	Yes	Yes
	80%	8/10	89%	8	2	10	Yes	No
	50%	5/10	25%	5	5	10	Yes	No
	90%	9/10	99%	9	1	10	No	No
	50%	5/10	25%	5	5	10	No	No
	60%	6/10	46%	6	4	10	No	No
	80%	8/10	89%	8	2	10	Yes	No
	70%	7/10	68%	7	3	10	Yes	No
	90%	9/10	99%	9	1	10	Yes	Yes
	80%	8/10	89%	8	2	10	Yes	Yes
	60%	6/10	46%	6	4	10	Yes	No
	80%	8/10	89%	8	2	10	Yes	Yes
	50%	5/10	25%	5	5	10	No	No
	70%	7/10	68%	7	3	10	Yes	No
	80%	8/10	89%	8	2	10	No	No
	80%	8/10	89%	8	2	10	Yes	No
	90%	9/10	99%	9	1	10	Yes	Yes
	70%	7/10	68%	7	3	10	Yes	Yes
	80%	8/10	89%	8	2	10	Yes	No
	70%	7/10	68%	7	3	10	Yes	Yes
	60%	6/10	46%	6	4	10	Yes	No
	50%	5/10	25%	5	5	10	Yes	No
	80%	8/10	89%	8	2	10	Yes	No
	50%	5/10	25%	5	5	10	No	No
	60%	6/10	46%	6	4	10	Yes	No
	60%	6/10	46%	6	4	10	No	No
	40%	4/10	10%	4	6	10	No	No
	80%	8/10	89%	8	2	10	Yes	No
	90%	9/10	99%	9	1	10	No	No
	40%	4/10	10%	4	6	10	No	No
	50%	5/10	25%	5	5	10	Yes	No
	50%	5/10	25%	5	5	10	Yes	No
	70%	7/10	68%	7	3	10	Yes	No
	100%	10/10	100%	10	0	10	Yes	Yes
	80%	8/10	89%	8	2	10	Yes	No
	80%	8/10	89%	8	2	10	Yes	No
	50%	5/10	25%	5	5	10	Yes	No
	40%	4/10	10%	4	6	10	No	No
	60%	6/10	46%	6	4	10	Yes	No

Respondent ID	iz Summary			Quiz Results				
	Score	Points	Percentile	Correct	Incorrect	Total_Questions	Do you know what is ultra-processed food?	Did you already know this definition?
	40%	4/10	10%	4	6	10	Yes	No
	80%	8/10	89%	8	2	10	Yes	No
	90%	9/10	99%	9	1	10	Yes	No
	80%	8/10	89%	8	2	10	Yes	No
	60%	6/10	46%	6	4	10	Yes	No
	80%	8/10	89%	8	2	10	Yes	Yes
	60%	6/10	46%	6	4	10	Yes	No
	60%	6/10	46%	6	4	10	Yes	No
	80%	8/10	89%	8	2	10	Yes	No
	70%	7/10	68%	7	3	10	Yes	No
	50%	5/10	25%	5	5	10	Yes	No
	90%	9/10	99%	9	1	10	No	No
	80%	8/10	89%	8	2	10	Yes	Yes
	80%	8/10	89%	8	2	10	Yes	No
	80%	8/10	89%	8	2	10	No	No
	30%	3/10	3%	3	7	10	No	No
	70%	7/10	68%	7	3	10	Yes	No
	80%	8/10	89%	8	2	10	No	No
	40%	4/10	10%	4	6	10	Yes	No
	70%	7/10	68%	7	3	10	Yes	Yes
	50%	5/10	25%	5	5	10	No	No
	70%	7/10	68%	7	3	10	Yes	No
	80%	8/10	89%	8	2	10	No	No
	70%	7/10	68%	7	3	10	Yes	No
	70%	7/10	68%	7	3	10	Yes	No
	70%	7/10	68%	7	3	10	Yes	No
	70%	7/10	68%	7	3	10	Yes	Yes
	60%	6/10	46%	6	4	10	Yes	No
	60%	6/10	46%	6	4	10	Yes	No
	80%	8/10	89%	8	2	10	Yes	No
	90%	9/10	99%	9	1	10	Yes	No
	70%	7/10	68%	7	3	10	Yes	No
	70%	7/10	68%	7	3	10	No	No
	30%	3/10	3%	3	7	10	Yes	No
	50%	5/10	25%	5	5	10	Yes	No
	80%	8/10	89%	8	2	10	No	No
	60%	6/10	46%	6	4	10	Yes	Yes
	90%	9/10	99%	9	1	10	Yes	No
	70%	7/10	68%	7	3	10	Yes	Yes
	100%	10/10	100%	10	0	10	Yes	No
	50%	5/10	25%	5	5	10	Yes	No
	50%	5/10	25%	5	5	10	Yes	No
	80%	8/10	89%	8	2	10	Yes	No
	50%	5/10	25%	5	5	10	Yes	No
	50%	5/10	25%	5	5	10	No	No

Table S1 Quiz Summary

**Respondent ID**	**iz Summary**			**Quiz Results**				
	**Score**	**Points**	**Percentile**	**Correct**	**Incorrect**	**Total_Questions**	**Do you know what is ultra-processed food?**	**Did you already know this definition?**
	60%	6/10	46%	6	4	10	No	No
	70%	7/10	68%	7	3	10	Yes	No
	80%	8/10	89%	8	2	10	Yes	No
	50%	5/10	25%	5	5	10	Yes	No
	90%	9/10	99%	9	1	10	Yes	Yes
	60%	6/10	46%	6	4	10	Yes	No
	50%	5/10	25%	5	5	10	Yes	No
	70%	7/10	68%	7	3	10	Yes	No
	80%	8/10	89%	8	2	10	Yes	Yes
	70%	7/10	68%	7	3	10	Yes	Yes
	80%	8/10	89%	8	2	10	Yes	Yes
	60%	6/10	46%	6	4	10	Yes	Yes
	80%	8/10	89%	8	2	10	Yes	No
	80%	8/10	89%	8	2	10	Yes	Yes
	60%	6/10	46%	6	4	10	Yes	Yes
	60%	6/10	46%	6	4	10	Yes	No
	80%	8/10	89%	8	2	10	Yes	No
	60%	6/10	46%	6	4	10	Yes	No
	80%	8/10	89%	8	2	10	Yes	Yes
	50%	5/10	25%	5	5	10	Yes	No
	50%	5/10	25%	5	5	10	Yes	No
	60%	6/10	46%	6	4	10	Yes	No
	80%	8/10	89%	8	2	10	Yes	No
	80%	8/10	89%	8	2	10	Yes	No
	70%	7/10	68%	7	3	10	Yes	No
	60%	6/10	46%	6	4	10	Yes	Yes
	70%	7/10	68%	7	3	10	Yes	No
	60%	6/10	46%	6	4	10	Yes	No
	80%	8/10	89%	8	2	10	No	No
	80%	8/10	89%	8	2	10	Yes	No
	60%	6/10	46%	6	4	10	Yes	No
	80%	8/10	89%	8	2	10	Yes	Yes
	80%	8/10	89%	8	2	10	No	No
	60%	6/10	46%	6	4	10	Yes	No
	80%	8/10	89%	8	2	10	Yes	No
	50%	5/10	25%	5	5	10	Yes	No
	80%	8/10	89%	8	2	10	Yes	Yes
	30%	3/10	3%	3	7	10	Yes	Yes
	70%	7/10	68%	7	3	10	Yes	No
	70%	7/10	68%	7	3	10	Yes	No
	80%	8/10	89%	8	2	10	Yes	No
	30%	3/10	3%	3	7	10	Yes	Yes
	60%	6/10	46%	6	4	10	No	No
	70%	7/10	68%	7	3	10	Yes	No
	40%	4/10	10%	4	6	10	Yes	No

Table S1 Quiz Summary

Respondent ID	iz Summary		Quiz Results					
	Score	Points	Percentile	Correct	Incorrect	Total_Questions	Do you know what is ultra-processed food?	Did you already know this definition?
	60%	6/10	46%	6	4	10	No	No
	90%	9/10	99%	9	1	10	Yes	No
	60%	6/10	46%	6	4	10	Yes	No
	50%	5/10	25%	5	5	10	Yes	No
	90%	9/10	99%	9	1	10	Yes	No
	50%	5/10	25%	5	5	10	Yes	Yes
	60%	6/10	46%	6	4	10	No	No
	40%	4/10	10%	4	6	10	Yes	No
	90%	9/10	99%	9	1	10	Yes	No
	50%	5/10	25%	5	5	10	Yes	No
	50%	5/10	25%	5	5	10	Yes	No
	90%	9/10	99%	9	1	10	Yes	No
	30%	3/10	3%	3	7	10	Yes	No
	90%	9/10	99%	9	1	10	No	No
	70%	7/10	68%	7	3	10	Yes	No
	60%	6/10	46%	6	4	10	Yes	No
	70%	7/10	68%	7	3	10	Yes	No
	40%	4/10	10%	4	6	10	Yes	No
	80%	8/10	89%	8	2	10	No	No
	60%	6/10	46%	6	4	10	Yes	Yes
	40%	4/10	10%	4	6	10	No	No
	70%	7/10	68%	7	3	10	Yes	No
	50%	5/10	25%	5	5	10	Yes	No
	100%	10/10	100%	10	0	10	Yes	No
	80%	8/10	89%	8	2	10	Yes	No
	80%	8/10	89%	8	2	10	Yes	No
	70%	7/10	68%	7	3	10	No	No
	70%	7/10	68%	7	3	10	Yes	No
	70%	7/10	68%	7	3	10	Yes	No
	60%	6/10	46%	6	4	10	Yes	No
	80%	8/10	89%	8	2	10	Yes	Yes
	70%	7/10	68%	7	3	10	No	No
	60%	6/10	46%	6	4	10	No	No
	50%	5/10	25%	5	5	10	Yes	Yes
	80%	8/10	89%	8	2	10	Yes	No
	60%	6/10	46%	6	4	10	Yes	No
	70%	7/10	68%	7	3	10	Yes	Yes
	70%	7/10	68%	7	3	10	Yes	No
	60%	6/10	46%	6	4	10	Yes	No
	80%	8/10	89%	8	2	10	Yes	Yes
	80%	8/10	89%	8	2	10	Yes	No
	50%	5/10	25%	5	5	10	No	No
	80%	8/10	89%	8	2	10	Yes	No
	40%	4/10	10%	4	6	10	No	No
	90%	9/10	99%	9	1	10	No	No

**Respondent ID**	**iz Summary**			**Quiz Results**				
	**Score**	**Points**	**Percentile**	**Correct**	**Incorrect**	**Total_Questions**	**Do you know what is ultra-processed food?**	**Did you already know this definition?**
	80%	8/10	89%	8	2	10	Yes	No
	80%	8/10	89%	8	2	10	No	No
	90%	9/10	99%	9	1	10	Yes	No
	90%	9/10	99%	9	1	10	Yes	No
	70%	7/10	68%	7	3	10	Yes	No
	50%	5/10	25%	5	5	10	No	No
	80%	8/10	89%	8	2	10	Yes	No
	70%	7/10	68%	7	3	10	Yes	No
	70%	7/10	68%	7	3	10	Yes	No
	70%	7/10	68%	7	3	10	No	No
	50%	5/10	25%	5	5	10	Yes	No
	70%	7/10	68%	7	3	10	Yes	No
	60%	6/10	46%	6	4	10	Yes	Yes
	50%	5/10	25%	5	5	10	Yes	No
	80%	8/10	89%	8	2	10	Yes	No
	80%	8/10	89%	8	2	10	Yes	No
	70%	7/10	68%	7	3	10	Yes	Yes
	90%	9/10	99%	9	1	10	Yes	No
	80%	8/10	89%	8	2	10	Yes	No
	80%	8/10	89%	8	2	10	Yes	Yes
	90%	9/10	99%	9	1	10	No	No
	70%	7/10	68%	7	3	10	Yes	No
	70%	7/10	68%	7	3	10	Yes	No
	70%	7/10	68%	7	3	10	Yes	Yes
	60%	6/10	46%	6	4	10	Yes	No
	60%	6/10	46%	6	4	10	Yes	No
	30%	3/10	3%	3	7	10	Yes	No
	70%	7/10	68%	7	3	10	Yes	No
	80%	8/10	89%	8	2	10	Yes	Yes
	60%	6/10	46%	6	4	10	Yes	Yes
	60%	6/10	46%	6	4	10	Yes	No
	90%	9/10	99%	9	1	10	Yes	No
	60%	6/10	46%	6	4	10	Yes	No
	70%	7/10	68%	7	3	10	No	No
	60%	6/10	46%	6	4	10	Yes	No
	80%	8/10	89%	8	2	10	Yes	No
	60%	6/10	46%	6	4	10	No	No
	70%	7/10	68%	7	3	10	Yes	No
	60%	6/10	46%	6	4	10	Yes	No
	80%	8/10	89%	8	2	10	Yes	No
	50%	5/10	25%	5	5	10	Yes	No
	40%	4/10	10%	4	6	10	Yes	Yes
	60%	6/10	46%	6	4	10	Yes	No
	60%	6/10	46%	6	4	10	Yes	No
	60%	6/10	46%	6	4	10	Yes	No

Respondent ID	iz Summary			Quiz Results				
	Score	Points	Percentile	Correct	Incorrect	Total_Questions	Do you know what is ultra-processed food?	Did you already know this definition?
	80%	8/10	89%	8	2	10	No	No
	80%	8/10	89%	8	2	10	No	No
	100%	10/10	100%	10	0	10	No	No
	100%	10/10	100%	10	0	10	No	No
	50%	5/10	25%	5	5	10	No	No
	50%	5/10	25%	5	5	10	Yes	Yes
	50%	5/10	25%	5	5	10	Yes	No
	50%	5/10	25%	5	5	10	Yes	Yes
	70%	7/10	68%	7	3	10	Yes	Yes
	90%	9/10	99%	9	1	10	No	No
	70%	7/10	68%	7	3	10	Yes	No
	60%	6/10	46%	6	4	10	No	No
	60%	6/10	46%	6	4	10	Yes	No
	70%	7/10	68%	7	3	10	No	No
	80%	8/10	89%	8	2	10	Yes	Yes
	70%	7/10	68%	7	3	10	Yes	No
	70%	7/10	68%	7	3	10	Yes	No
	60%	6/10	46%	6	4	10	Yes	Yes
	80%	8/10	89%	8	2	10	Yes	Yes
	60%	6/10	46%	6	4	10	Yes	No
	50%	5/10	25%	5	5	10	Yes	No
	50%	5/10	25%	5	5	10	Yes	Yes
	60%	6/10	46%	6	4	10	No	No
	50%	5/10	25%	5	5	10	No	No
	80%	8/10	89%	8	2	10	Yes	No
	70%	7/10	68%	7	3	10	Yes	No
	80%	8/10	89%	8	2	10	Yes	Yes
	50%	5/10	25%	5	5	10	Yes	No
	40%	4/10	10%	4	6	10	Yes	No
	70%	7/10	68%	7	3	10	Yes	No
	70%	7/10	68%	7	3	10	No	No
	60%	6/10	46%	6	4	10	Yes	No
	60%	6/10	46%	6	4	10	No	Yes
	80%	8/10	89%	8	2	10	Yes	No
	70%	7/10	68%	7	3	10	Yes	No
	70%	7/10	68%	7	3	10	Yes	No
	80%	8/10	89%	8	2	10	Yes	No
	60%	6/10	46%	6	4	10	Yes	No
	60%	6/10	46%	6	4	10	Yes	No
	70%	7/10	68%	7	3	10	Yes	Yes
	80%	8/10	89%	8	2	10	Yes	No
	80%	8/10	89%	8	2	10	Yes	No
	80%	8/10	89%	8	2	10	Yes	No
	70%	7/10	68%	7	3	10	Yes	No
	50%	5/10	25%	5	5	10	Yes	No

Table S1 Quiz Summary

Respondent ID	iz Summary		Quiz Results					
	Score	Points	Percentile	Correct	Incorrect	Total_Questions	Do you know what is ultra-processed food?	Did you already know this definition?
	70%	7/10	68%	7	3	10	Yes	No
	60%	6/10	46%	6	4	10	Yes	No
	70%	7/10	68%	7	3	10	Yes	No
	60%	6/10	46%	6	4	10	Yes	No
	70%	7/10	68%	7	3	10	Yes	No
	50%	5/10	25%	5	5	10	Yes	No
	60%	6/10	46%	6	4	10	Yes	No
	50%	5/10	25%	5	5	10	Yes	No
	90%	9/10	99%	9	1	10	Yes	No
	80%	8/10	89%	8	2	10	Yes	No
	80%	8/10	89%	8	2	10	Yes	Yes
	80%	8/10	89%	8	2	10	No	No
	60%	6/10	46%	6	4	10	Yes	No
	40%	4/10	10%	4	6	10	Yes	Yes
	70%	7/10	68%	7	3	10	Yes	No
	60%	6/10	46%	6	4	10	Yes	No
	50%	5/10	25%	5	5	10	Yes	No
	70%	7/10	68%	7	3	10	Yes	No
	70%	7/10	68%	7	3	10	Yes	Yes
	90%	9/10	99%	9	1	10	Yes	No
	50%	5/10	25%	5	5	10	Yes	Yes
	70%	7/10	68%	7	3	10	Yes	No
	50%	5/10	26%	5	5	10	Yes	Yes
	90%	9/10	99%	9	1	10	Yes	Yes
	60%	6/10	46%	6	4	10	No	No
	80%	8/10	89%	8	2	10	No	No
	40%	4/10	10%	4	6	10	Yes	No
	50%	5/10	26%	5	5	10	Yes	No
	90%	9/10	99%	9	1	10	Yes	No
	50%	5/10	26%	5	5	10	Yes	No
	80%	8/10	89%	8	2	10	Yes	No
	60%	6/10	46%	6	4	10	Yes	Yes
	70%	7/10	68%	7	3	10	Yes	No
	60%	6/10	46%	6	4	10	Yes	No
	60%	6/10	46%	6	4	10	Yes	No
	50%	5/10	26%	5	5	10	No	Yes
	70%	7/10	68%	7	3	10	No	No
	80%	8/10	89%	8	2	10	Yes	No
	80%	8/10	89%	8	2	10	Yes	No
